# Half a century of coastal temperature records reveal complex warming trends in western boundary currents

**DOI:** 10.1038/s41598-017-14944-2

**Published:** 2017-11-06

**Authors:** Nick T. Shears, Melissa M. Bowen

**Affiliations:** 1 Leigh Marine Laboratory, Institute of Marine Science, University of Auckland, Auckland, New Zealand; 2 School of Environment, University of Auckland, Auckland, New Zealand

## Abstract

Accelerated warming of western boundary currents due to the strengthening of subtropical gyres has had cascading effects on coastal ecosystems and is widely expected to result in further tropicalization of temperate regions. Predicting how species and ecosystems will respond requires a better understanding of the variability in ocean warming in complex boundary current regions. Using three ≥50 year temperature records we demonstrate high variability in the magnitude and seasonality of warming in the Southwest Pacific boundary current region. The greatest rate of warming was evident off eastern Tasmania (0.20 °C decade^−1^), followed by southern New Zealand (0.10 °C decade^−1^), while there was no evidence of annual warming in northeastern New Zealand. This regional variability in coastal warming was also evident in the satellite record and is consistent with expected changes in regional-scale circulation resulting from increased wind stress curl in the South Pacific subtropical gyre. Warming trends over the satellite era (1982–2016) were considerably greater than the longer-term trends, highlighting the importance of long-term temperature records in understanding climate change in coastal regions. Our findings demonstrate the spatial and temporal complexity of warming patterns in boundary current regions and challenge widespread expectations of tropicalization in temperate regions under future climate change.

## Introduction

The redistribution of species due to human mediated climate change is having increasing impacts on ecosystems and human wellbeing^1^. Poleward shifts of species are widely expected in response to warming of the oceans^2^, and this is expected to lead to the “tropicalization” (increased proportion of tropical species) of temperate regions^3^. Some of the clearest examples of tropicalization come from western boundary current regions^4^, where accelerated warming has occurred due to the strengthening of subtropical gyres^5^. This has had cascading effects on coastal ecosystems in some temperate regions^4,6^. However, there is increasing awareness that warming trends in the oceans are not uniform and species responses closely track the complex mosaic of local climate velocities^7^. Therefore, better understanding of how temperatures are changing, both temporally and spatially in the coastal environment, is crucial for predicting how species and ecosystems will be affected by climate change.

Much of our understanding of long-term changes in ocean temperatures are based on analyses of reconstructed global sea surface temperature (SST) data sets^5,8–10^. Despite uncertainties associated with limited sampling, changing techniques and analytical procedures, there is generally a high degree of concordance in large-scale global patterns in warming among datasets^10^. However, such datasets are most suitable for long-term global and basin-wide studies rather than assessing changes in complex coastal environments^11,12^. The availability of satellite-derived estimates of SST over large spatial scales for the last 3-4 decades has led to major advances in the analysis and understanding of long-term trends in sea surface temperature globally^13^. However, the relatively short duration of available time series from satellites severely limits their application for detecting long-term trends in SST^14^. Such trends are sensitive to the time-span over which they are computed, and may be strongly influenced by multi-year climate cycles, such as the El Niño Southern Oscillation (ENSO)^15^. Consequently, long-term *in situ* SST records, with regular standardised measurements spanning longer time periods, are extremely valuable for detecting longer-term trends in SST and evaluating trends reported from other SST data sources, particularly in coastal waters. Such records are generally rare, but in some cases date back more than 100 years^16–20^.

**Figure 1:**
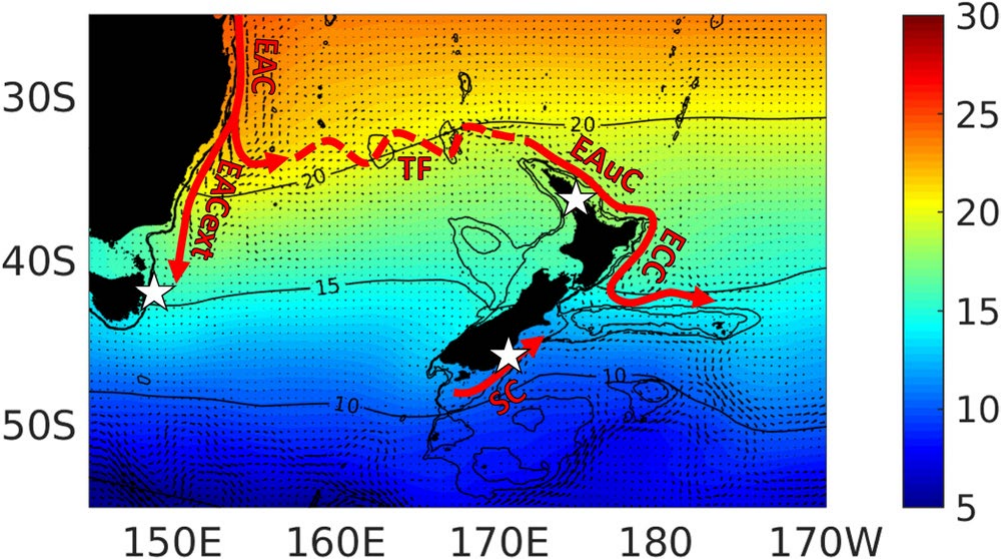
Mean sea surface temperature (^o^C; NOAA OISST v2 from 1982–2016), major subtropical boundary currents (red arrows) and dominant surface currents (small black arrows; mean absolute geostrophic velocities from CLS-CNES distributed by Aviso*) in the Southwest Pacific boundary current region and the locations of long-term monitoring stations (Stars indicate coastal SST stations: Maria Island eastern Tasmania, Leigh Marine Laboratory northeastern New Zealand and Portobello Marine Laboratory southeastern New Zealand). EAC: East Australian Current and extension (EACext); TF: Tasman Front; EAuC: East Auckland Current; ECC: East Cape Current; SC: Southland Current. Bathymetric contours are 500 m and 1000 m. See Fig. S1 for comparison of seasonal cycle in sea surface temperature between the three stations. Map produced in Matlab R2017a, https://au.mathworks.com/products/matlab.html. *http://www.aviso.altimetry.fr/en/data/products/sea-surface-height-products/global/madt.html.

The long-term SST record from Maria Island, eastern Tasmania, has been pivotal in documenting and understanding the dynamics of warming that has occurred off the east coast of Tasmania, Australia, over the last 6–7 decades^8,21^. This warming has been attributed to the intensification and pole-ward extension of the East Australian Current (EAC; Fig. 1)^8^, resulting from increasing wind stress curl at mid-latitudes causing a strengthening of the South Pacific Ocean subtropical gyre^5^. Warming in this region has resulted in large-scale changes in coastal ecosystems^6,22^ and similar changes are widely predicted in boundary current regions worldwide^4^. However, the generality and wider occurrence of warming in this boundary current region has not been examined and it is unclear whether the ecological changes observed may be expected elsewhere. Boundary current regions are often characterised by complex current systems^23^ and climate-related changes in gyres may differentially affect the strength of major currents and result in variable warming trends across a region. Understanding this variability is essential to predicting how species and ecosystems will respond regionally to these large-scale oceanographic changes.

In this study we use three of the longest sea surface temperature records in the Southern Hemisphere (50–71 years) to investigate trends, and seasonality of trends, in coastal temperatures within the Southwest Pacific boundary current region. The three SST records examined, from eastern Tasmania (Maria Island), and northern (Leigh) and southern (Portobello) New Zealand, span this complex boundary current region and are differentially influenced by the major currents within the region (Fig. 1). Maria Island is influenced by the East Australian Current (EAC) extension, which brings southward flowing subtropical waters from the EAC^8^. The EAC is the dominant boundary current and most of this current separates from the east Australian coast between ~28 and 33°S^24^ and flows eastward into the Tasman Sea, where it forms the Tasman Front^25^. Some of this water ultimately feeds into the East Auckland Current (EAuC) that flows down the northeast continental margin of New Zealand^26^ (Fig. 1). It may therefore be expected that warming of the EAC could translate into warming in the EAuC with resulting effects on species distributions in northeastern New Zealand^4,27,28^. Conversely, it has been demonstrated that stronger wind stress curl over the south Pacific favours the EAC extension pathway, at the expense of the Tasman Front, whereby more warm water from the EAC is transported into the southern Tasman Sea^29^. Subtropical waters from the southern Tasman flow around the southern tip of the South Island as part of the Southland Current^30,31^ providing a potential mechanism for warming around southern New Zealand.

We compare trends in SST anomalies among these three locations over the last half century to investigate how large-scale changes in the South Pacific Ocean subtropical gyre have affected coastal SST in this region. To determine the primary drivers of long-term change in SST we analyse the relationships between the SST records and a suite of meteorological and climatic indices. We also investigate the coherence between the coastal SST records and satellite-derived SST data, and compare observed trends at the coastal stations with trends across the region. This study highlights the spatial and temporal complexity of warming patterns in boundary current regions and provides a basis for predicting ecological change across the region. It also emphasises the importance of long-term temperature records in documenting, validating and understanding long-term changes in SST in coastal waters.

## Results

**Figure 2:**
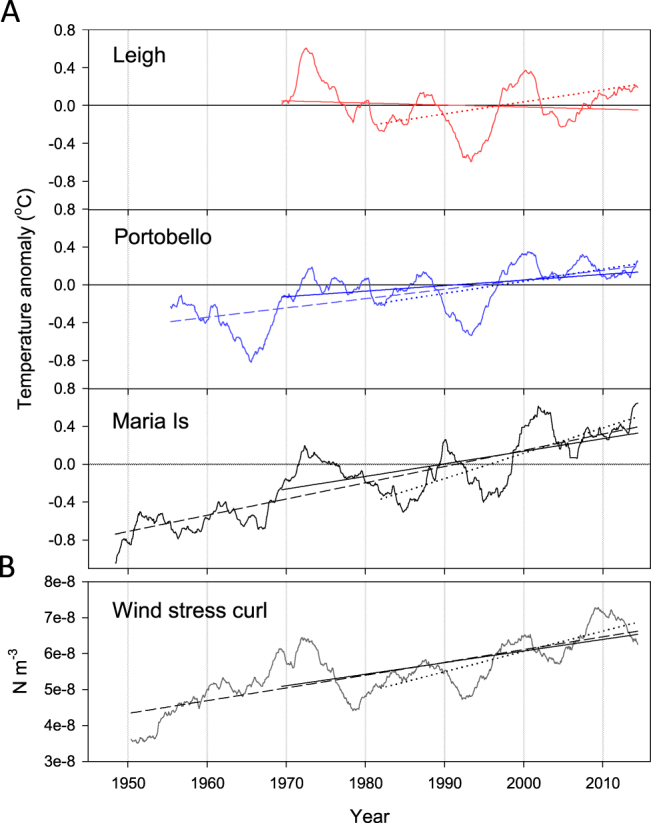
Long-term trends in monthly sea surface temperature anomaly at coastal stations (**A**) and South Pacific regional mean wind stress curl (**B**). Data are low-pass filtered using a 5-year running mean to highlight low frequency variability. Solid lines show linear trend over the last 50 years, dotted line shows trend over the satellite era (1982–2016), dashed line shows the trend for the full time series at Portobello and Maria Island. Wind stress curl was calculated over the region 180–280°E and 20–50°S.

The three coastal stations examined had varying long-term trends in monthly SST anomalies (Fig. 2A, Table 1A). The greatest warming trend was evident at Maria Island (+0.20 °C decade^−1^ from 1946–2016), followed by Portobello (+0.10 °C decade^−1^ from 1953–2016), and no trend was evident at Leigh over the last 50 years. When analyses were restricted to the last 50 years, to provide a more direct comparison with the time series at Leigh, the warming trends were marginally lower at Maria Island and Portobello (+0.16 and +0.08 °C decade^−1^ respectively) (Table 1B). While the long-term trends differ among the three locations, they do show similar decadal variation in SST (Fig. 2A). Cross correlation analysis on detrended annual SST indicated that SST was most strongly correlated between Leigh and Portobello at lag zero (r = 0. 80). Leigh and Maria Island had the strongest correlation at lag zero (r = 0.44), but there was also evidence that annual temperatures at Maria Island lagged those at Leigh by 2–4 years (r = 0.33–0.34). Annual temperatures at Maria Island lagged Portobello by three years (r = 0.48) (Table S1).

**Table 1:** Regression analysis of long-term trends in monthly SST anomalies from the coastal SST records (**A–C**) and satellite derived SST for offshore stations each region (**D**).

	t-statistic	*p*-value	Trend °C decade ± 95%CI
**(A) Full time series**			
Leigh (1967–2016)	0.43	0.666	0.021 ± 0.094
Portobello (1953–2016)	**4.16**	<**0.001**	**0.100** ± **0.047**
Maria Is (1946–2016)	**7.62**	<**0.001**	**0.200** ± **0.051**
**(B) 1967–2016**			
Portobello	**2.46**	**0.014**	**0.079** ± **0.063**
Maria Is	**3.86**	<**0.001**	**0.161** ± **0.082**
**(C) 1982–2016**			
Leigh	1.66	0.098	0.122 ± 0.144
Portobello	**2.68**	**0.008**	**0.147** ± **0.107**
Maria Is	**4.54**	<**0.001**	**0.319** ± **0.138**
**(D) Satellite-derived SST (1982–2016)**			
Northeastern NZ	1.144	0.253	0.080 ± 0.138
Southeastern NZ	**2.990**	**0.003**	**0.234** ± **0.153**
Eastern Tasmania	**3.274**	**0.001**	**0.298** ± **0.178**

Trends are calculated over different time periods to allow comparison between the data sets of differing lengths. Bold indicates significant trends.

**Figure 3:**
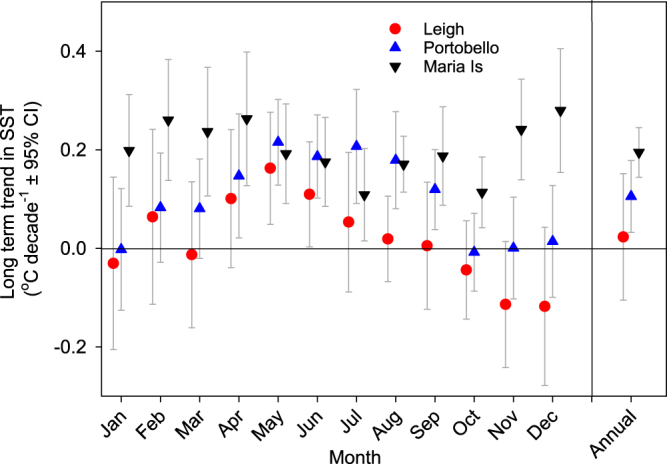
Seasonal variability in long-term change in sea surface temperature across the entire time series at each coastal station (confidence intervals that overlap with zero are not significant (α = 0.05)). Slopes are based on linear regression of mean monthly SST anomalies analysed separately for each month over the time series. The slope coefficient based on analysis of annual mean SST anomaly is also shown. When the equivalent years to the Leigh time series were analysed (1967–2016) for Portobello and Maria Island, the seasonal variability in warming and slope coefficients were largely consistent with that seen in the full time series (Fig. S4).

Trends in temperature by month exhibited considerable seasonal differences in warming trends among the three coastal stations. Long-term warming was evident across all months at Maria Island (+0.11–0.28 °C decade^−1^, Fig. 3). At Portobello significant warming was only evident from April-September (+0.12–0.22 °C decade^−1^). At Leigh, significant warming was evident in May and June (+0.16 and +0.11 °C decade^−1^), and there was some indication of a negative trend in SST anomaly for the months of November and December.

**Figure 4:**
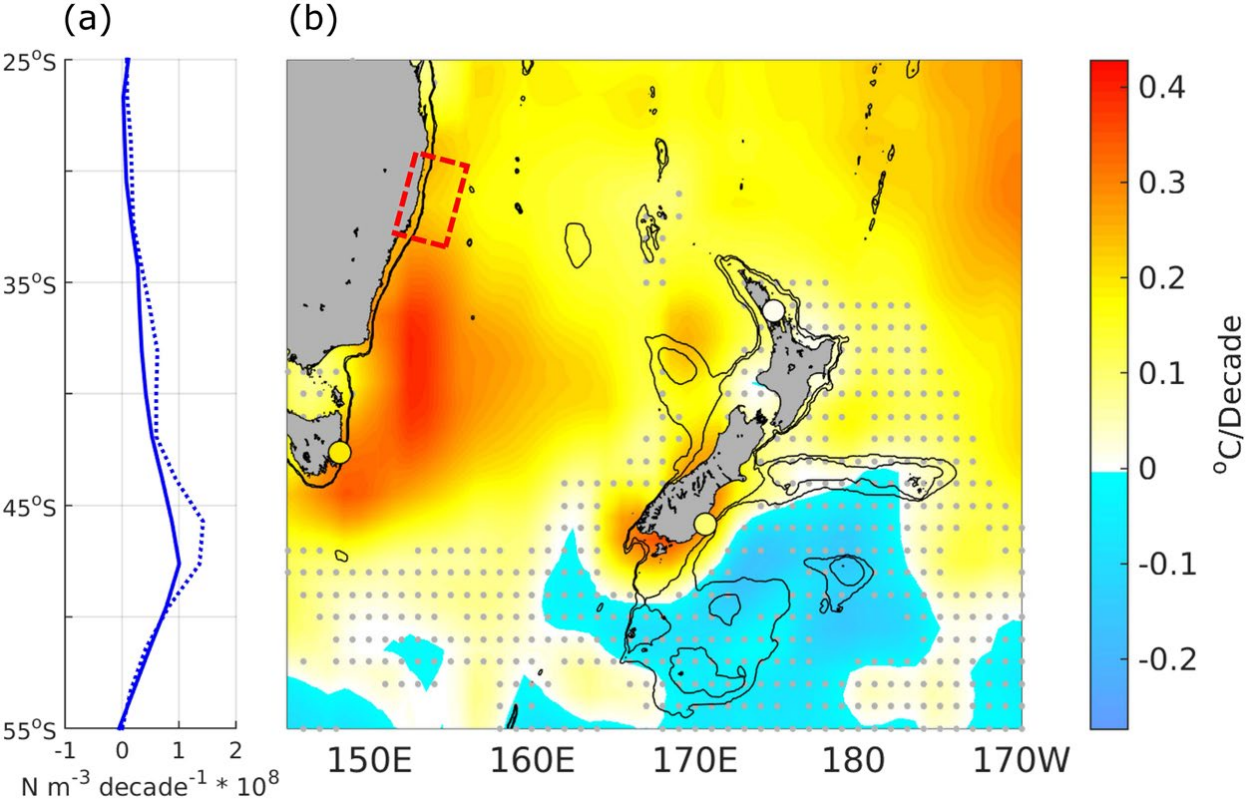
Trends in wind stress curl with latitude (**A**: 1948–2016 solid line; 1982–2016 dashed line) and region-wide trends in sea surface temperature based on satellite-derived SST (OISST v2) from 1982–2016 (**B**). Red box shows area of the EAC separation^24^, small grey dots denote locations with non-significant trends in SST. Large coloured dots show the three coastal SST stations with colour indicating long-term trend (°C decade^−1^) in SST at each. Bathymetric contours are 500 m and 1000 m. Trends in wind stress curl were calculated over the region 180–280°E. Map produced in Matlab R2017a, https://au.mathworks.com/products/matlab.html.

Wind stress curl over the South Pacific increased over the last half century with interannual variations similar to those seen in the SST records (Fig. 2). Increases in wind stress curl were greater at southern latitudes of the South Pacific (Fig. 4A), and would be expected to increase the wind-driven circulation in the southern latitudes of the South Pacific subtropical gyre (Fig. S2). Inter-annual variation in SST at all stations was most strongly correlated with wind stress curl at lag zero, but there was also significant correlation at lag one year for Leigh and Portobello, and lag 3 and 5 years at Maria Island (Table S1).

Inter-annual variation in SST was not clearly related to the southern annular mode (SAM), but was significantly correlated with the Southern Oscillation Index (SOI) at all stations at lag zero (Table S1), with highest correlation at Leigh. The SOI did not have a significant trend over the full monitoring period that might lead to a trend in SST, but there has been a marginally significant (p = 0.071) trend in the SOI over the satellite era (Fig. S3, Table S1).

Monthly anomalies in the coastal SST records were strongly correlated with those from satellite-derived SST at offshore stations in each region (OISST v2) (Leigh: *r* = 0.81; Portobello: *r* = 0.69, Maria: *r* = 0.66). Trends in satellite-derived SST were consistent with those seen in the coastal records from 1982–2016 (Fig. S5 and Table 1C and D), with the greatest warming off eastern Tasmania, followed by southeastern NZ and a non-significant warming trend in northeastern NZ. However, warming trends over the satellite era were considerably greater than those seen over the full time period for each of the long-term records (Fig. 2A, Table 1A and C). Satellite-derived SST in each region exhibited analogous seasonal variation in warming trends to those in the coastal records over the satellite era (1982–2016: Fig. S6 and Table 1)).

Trends in satellite-derived SST revealed large-scale warming off the east coast of Australia south of the EAC separation and in subtropical waters to the northeast of New Zealand, and some regions of cooling southeast of New Zealand (Fig. 4B). There was no significant trend around the northern coast of New Zealand, but significant warming around the southern coast of New Zealand.

## Discussion

The long-term temperature records examined in this study demonstrated high spatial and temporal variability in warming trends in the SW Pacific boundary current region over the last half century. The previously well documented warming off eastern Tasmania^21^ has not occurred at the same magnitude in waters surrounding New Zealand, and unlike off Tasmania, the warming trends in New Zealand waters are strongly seasonally-dependent. The differing trends among the three coastal stations examined are consistent with the changes in circulation associated with the spin-up of the South Pacific subtropical gyre^21,29,32,33^. The increase in wind stress curl is largely apparent south of the separation region of the EAC (Fig. 4A), leading to increased flows in the southern latitudes of the subtropical gyre (Fig. S2). The associated increases in the flow of boundary currents south of the separation region are predicted to continue^32^. The two southern-most stations, Maria Island and Portobello, are influenced by the EAC extension and Southland Current respectively and have exhibited significant warming trends, which is consistent with increased flows in these boundary currents. In contrast, there was no evidence of long-term increases in annual temperatures at the northern New Zealand station (Leigh), which is influenced by the East Auckland Current that originates at similar latitudes to the EAC separation.

The spatially variable long-term trends observed in the coastal SST records were reflected in the analysis of regional-scale trends in warming over the satellite era (1982–2016) based on the OISST product, although warming trends over this period were considerably greater than those seen over the full time series (discussed below). The OISST indicated considerable warming off the east Australian coast south of the EAC separation and extending across much of the Tasman Sea, and in coastal waters around most of the South Island of New Zealand. There was no indication of large-scale warming in coastal waters around the North Island of New Zealand, including the offshore regions of the East Auckland and East Cape current’s east of northern New Zealand. The differing trends in SST between the Tasman Sea and eastern New Zealand are consistent with changes in circulation under increasing wind stress curl with increased warm water flows into the Tasman and reduced flows into the Tasman Front^29,33^. These trends contrast those previously reported at several coastal locations around New Zealand based on analysis of the ERSST v.3b globally reconstructed SST^34^. Examination of regional trends in the two globally reconstructed SST products, ERSST v.4 and HadISST (Fig. S7), demonstrate how these products do not capture the complex variation in trends in coastal waters. Both indicated stronger warming in northern New Zealand and no warming in the south, the opposite to trends observed in the *in-situ* records and the OISST. These findings reinforce that these global-scale SST data products are more suited to basin-scale analyses^11,12^.

The long-term warming trend at Portobello and the broader-scale warming around the southern coast of New Zealand (shown in the OISST) are consistent with the overall increase in temperatures in boundary currents at the poleward edges of all the subtropical gyres associated with systematic changes in the winds^5^. While more research is needed to better understand the exact mechanisms driving warming in this region, the narrow region of high SST trends around the southern coast of the South Island suggest that the warming is associated with the subtropical front. The subtropical front is found near the 500 metre isobath along the continental shelf and can extend south to nearly 49°S on the shallow Snares shelf south of New Zealand^30^. Transport of subtropical water from the Tasman to the Pacific along the Otago coast is small (~1 Sv) but persistent^31^, although the dynamics that control the current are not well understood^35^. Increased temperatures around southern New Zealand may be due to both increased temperatures in the Tasman Sea and increased flow in the Southland Current. Paleoceanographic estimates of temperature from a previous interglacial warm period also show SST around southern New Zealand concurrent with warmer temperatures south of Tasmania and cooler temperatures in the EAuC^36^.

Interannual variations in SST at all three coastal stations were significantly correlated with interannual variations in South Pacific wind stress curl, with highest correlations at no time lag (Fig. 3 and Table S1). Lagged correlations have been used previously to infer that SST at Maria Island varies in response to flow in the EAC extension, with the three year delay due to the propagation of the wind signal as baroclinic Rossby waves across the Tasman Sea^21^. Here we find the highest correlation of SST and wind stress curl at no lag for all the stations, which suggests several possible alternative mechanisms. The correspondence at no lag may be the result of rapid barotropic adjustment, with years of stronger wind stress curl creating years with stronger flow in the boundary currents. Alternatively, upper ocean temperatures may be influenced by subsurface temperatures and the adjustment of the thermocline to changes in the wind stress curl^37^, variability of air-sea heat flux^37^ or baroclinic instability of the Tasman Sea thermocline^33^. Although, the mechanism causing interannual temperature changes has yet to be determined, the significant correlation at all the stations over the last half century suggests that SST is likely to vary with South Pacific wind stress curl in the future.

Strong seasonal variation in the magnitude and occurrence of warming was evident at the two New Zealand stations. At Leigh there was evidence of long-term warming in May and June, and some indication of a decline in temperatures in November and December. This may reflect a long-term shift in seasonality and suggests that factors besides advection by ocean currents may influence long-term changes in temperature in this region. At Portobello there was an increase in autumn-winter temperatures of 1.3 °C (0.15–0.22 °C decade^−1^) but no long-term change in summertime temperatures. Similar seasonal trends were evident in the satellite data offshore from Portobello (Table S1) indicating this is likely related to large-scale oceanographic rather than terrestrial or meteorological influences. These seasonal patterns contrast with those from Maria Island where there was no clear seasonal variation in warming trends. One possibility is that warming surface temperatures during autumn-winter at Portobello reflect an underlying increase in deeper subtropical water temperatures that are masked during summer months. Similar mechanisms explain wintertime warming trends in parts of the Gulf of Mexico^38^, when the warmer waters of the Loop Current have a greater influence on surface water temperatures in the region. Given the fundamental importance of seasonal variation in temperature for biological processes^39^, the mechanisms responsible for seasonal variability in warming in both northern and southern New Zealand deserve further investigation.

We found strong coherence between the inshore SST datasets and satellite-derived SST (OISST v2) from adjacent offshore locations, indicating that (1) long-term temperature measurements from these coastal locations reflect larger-scale oceanographic processes, and (2) satellite-derived SST measurements are useful as a proxy for examining large-scale trends in coastal SST. However, rates of warming over the satellite era were considerably greater than rates of warming over the full monitoring period (50–71 years) at each long-term station. This higher rate of warming is coincident with the positive trend in SOI and a steeper trend in wind stress curl over the satellite era (Table S2). At all locations annual SST was positively correlated with SOI, with cooler annual temperatures during El Niño (negative SOI) and warmer temperatures during La Niña (positive SOI) as seen in previous studies^40^. The dominant ENSO phase has changed over the satellite period, with El Niño’s predominating in the 1980s and early 1990s, and more La Niña conditions predominating in the 2000s (Fig. S3). Therefore, the steeper trends in SST over the satellite era compared to the longer-term trends are related to fluctuations in ENSO over this period (Fig. 4B). In contrast, there was no evidence of a long-term trend in SOI over the full time period of the coastal SST records (Fig. 2, Table S2), indicating that fluctuations in ENSO are not likely to have influenced the longer-term trends observed. Our results demonstrate how the presence and strength of trends in SST can be greatly influenced by the length of time over which data are analysed (Fig. 2A) and reiterate the need to take into account climate cycles such as ENSO when analysing shorter-term SST records.

Climate simulations project increases in the EAC flow, in particular south of the separation in the EAC extension^32,41,42^. Thus, the temperature trends identified in our study should continue if trends in the wind stress curl continue. The previously documented warming trend in coastal waters off eastern Australia has led to numerous ecological changes due to the arrival of warmer-water species historically found in northern waters^4,43,44^. The trends in SST reported from coastal waters around New Zealand highlight the variability in warming trends that can occur in boundary current regions and indicate that similar patterns of tropicalisation may not be universal. The lack of widespread warming in coastal waters around northern New Zealand substantially reduces the potential for increases in tropical and subtropical species in this region. Furthermore, the continuing intensification of the EAC and weakening of the Tasman Front is predicted to considerably reduce the connectivity between East Australia and northern New Zealand^45^. This could in fact lead to declines in populations of tropical and subtropical species that are currently sustained by larval transport across the Tasman Sea. Continued warming in southern parts of New Zealand may also result in the loss of cooler water species that are geographically constrained from spreading further south. A reduced thermal gradient across New Zealand combined with reduced connectivity to populations northwest of New Zealand could potentially lead to biotic homogenisation of coastal ecosystems, a phenomenon that has been documented in terrestrial systems as a response to climate change^46,47^. This would be in stark contrast to patterns of tropicalisation that are broadly predicted for boundary current regions worldwide^4^.

The Southwest Pacific boundary current region has been identified as an area of profound changes in circulation as a result of climate change^45^ and our study demonstrates how this can translate into complex and variable warming trends in coastal waters. Similar variability in warming trends may be expected in other boundary current regions where major circulation changes are predicted with climate change. The spatial and seasonal variability in warming patterns pose a number of challenges in predicting how species and ecosystems will change in coastal waters in boundary current regions. Interdisciplinary studies combining physical oceanography and ecology are therefore essential to understanding the complexity of boundary currents, the mechanisms driving variation in warming patterns, and predicting how ecosystems will change in the future. Our study also highlights the immense value of long-term data sets for documenting and understanding climate change in coastal waters.

## Methods

### Long-term temperature records

Daily sea surface temperatures have been measured at the University of Auckland’s Leigh Marine Laboratory and the University of Otago’s Portobello Marine Laboratory since 1967 and 1953 respectively (Fig. 1). The Leigh Marine Laboratory is located on an exposed rocky coast in northeastern New Zealand, whereas the Portobello Marine Laboratory is located within the Otago Harbour, southeastern New Zealand. Daily measurements of surface water temperature (<2 m depth) are taken at approximately 9am from the rocky shore at Leigh 36°16.12′S; 174°48.01′E;^48^ and from a wharf at Portobello (45°49.68′S; 170°38.39′E). Measurements were automated at Leigh in October 2011, with deployment of a temperature logger ~1 m below the surface, and ~200 m offshore from the original site. Daily data from 9 am was used for comparison with previous data. Two gaps exist in the Leigh time series, May-September 2011 and March-June 2013. For the May-September 2011 period, monthly means for Leigh were estimated based on the relationship between monthly means at Leigh and monthly means from satellite (see next section) from 1982–2013; y = 1.1443x − 3.8228; R² = 0.9644. For the period from March-June 2013, daily temperature data were used from a site approximately 600 m to the west (36°15.96′S; 174°47.80′E). Mean monthly temperatures from this site are very similar to the historic site (for the period March 2012-Jan 2014: SST = 1.0532x − 0.9829; R² = 0.9992.

At Maria Island (42° 36′S, 148° 14′E) ship-based measurements of sea temperature have been taken approximately every 2–6 weeks since 1944. This station is located on the 50 m isobar ~8 km offshore from Maria Island and water temperature is measured throughout the water column. For consistency with temperature data from Leigh and Portobello, only surface temperature data (≤2 m depth) was used from Maria Island in this study. While higher frequency temperature data is also available at this station from a mooring (since 2009), analyses were restricted to the ship-based measurements for consistency throughout the sampling period. Temperature data from Maria Island were downloaded from the Australian Ocean Data Network. Data from 1944–2014 were extracted from the “IMOS - ANMN National Reference Stations - Combined long-term hydrological data product (1944–2014)” (Accessed: 27/04/2017), and data for 2015–2016 were extracted from “IMOS - Australian National Mooring Network (ANMN) - CTD Profiles” (Accessed: 27/04/2017).

### Analysis of long-term trends in sea surface temperature

Monthly SST anomalies were calculated for each station based on the long-term mean monthly temperatures from 1967–2016 to provide consistency across the three data sets. At Maria Island the mean monthly temperatures each year were typically based on 1 or 2 measurements a month (compared to daily measurements at Leigh and Portobello), and in most years measurements were not available for every month. The first two years (1944 and 1945) were excluded from the analysis as there were only two sampling occasions and including these values influenced the overall trend.

Long-term trends in the monthly SST anomaly was analysed for each station using Generalized Least Squares regression. This approach takes into account auto-correlation across years and therefore provides a more conservative estimate of whether trends are significant compared to linear regression. Model fitting was done using Restricted Maximum Likelihood (REML) and included an autoregressive process (AR1) for the residuals. To determine the potential influence of the shorter time series available at Leigh on reported trends, analyses on the Maria Island and Portobello data set were also carried out over the same time period as Leigh (1967–2016).

To examine seasonal variation in long-term trends, the SST anomaly was analysed over time for each month. Long-term variation in SST anomaly for a given month is less influenced by autocorrelation so a model comparison approach (AIC) was used for each monthly analysis to determine whether it was necessary to include the autoregressive process in the linear model. Analyses were also carried out on the long-term annual anomaly (i.e. average of monthly anomalies for each year). These analyses were repeated for the full and restricted (1967–2016) time series for Maria Island and Portobello.

### Climate observations

Trends in a number of key climatic variables were examined using the same approach as above. Wind stress curl ($\nabla x\tau$) for the South Pacific was calculated over the region 180–280°E and 20–50°S using monthly wind stress ($\tau$) from the NCEP-1 Reanalysis^49^. The trends in the wind stress curl averaged between 180–280°E were calculated at each latitude of the NCEP-1 Reanalysis for entire time series (1948–2016) and for the satellite era (1982–2016). The wind-driven flow was estimated using the Sverdrup balance^50^ $({\rm{\beta }}\,V=\frac{1}{{\rm{\rho }}}\nabla x{\rm{\tau }})$ and integrating the depth-averaged meridional flow (V) from South America to the western boundary to create a transport stream function $({\rm{\Psi }}=-\int V\,dx)$. Following the Island Rule^51^, we treat the submarine platform of New Zealand between 33^o^ S and 55^o^ S as an island and find the value of the stream function at the boundary. We use this boundary value to continue integrating the stream function from New Zealand across the Tasman Sea. At each location the mean and trend of the stream function were calculated from the JRA-55 wind stress^52^ from 1958 to 2016.

Trends in the following three climate indices were also analysed: the Southern Annular Mode (SAM; www.antarctica.ac.uk/met/gjma/sam.html), the Southern Oscillation Index (SOI; www.esrl.noaa.gov/psd/gcos_wgsp/Timeseries/SOI/) and the Pacific Decadal Oscillation Index (PDO; www.ncdc.noaa.gov/teleconnections/). Cross-correlation analysis between SST anomalies and climatic variables were carried out using detrended annual means.

### Comparison with satellite-derived SST

Remote-sensed monthly SST measurements were obtained for offshore stations in each of the three regions from NOAAs Optimum Interpolation Sea Surface Temperature (OISST) v2^13^. These data are available in 1° grid format from 1982–2016 and were extracted for the nearest available points to Leigh, Portobello and Maria Island (35.5°S 175.5°E, 45.5°S 171.5°E, −42.5°S, 148.5°E). Trends in satellite-derived SST were analysed using the same method as for coastal data records. Correlation coefficients were calculated between annual means from the satellite data and coastal SST records for each location.

### Regional analysis of SST trends

Regional variation in SST trends across the Southwest Pacific were analysed for the satellite-derived data set (OISST v2) and two global reconstructed SST datasets. Long-term reconstructed SST records were obtained from two sources: NOAAs Extended Reconstructed Sea Surface Temperature Version 4 (ERSST v.4) dataset^53^ and the Hadley centre Global sea-Ice coverage and Sea Surface Temperature (HadISST) v1.1 dataset^12^. For consistency this analysis was restricted to the satellite era (1982–2016). SST data from each data set were extracted for the area surrounding New Zealand and the Tasman Sea. The trend was calculated at each location between January 1982 and December 2016 after removing the mean seasonal cycle, with significance at the 95% level calculated from a standard t-test with degrees of freedom estimated from the autocorrelation functions of the detrended data^54^.

## Electronic supplementary material

Supplementary material
