# Supplementary material for: Half a century of coastal temperature records reveal complex warming trends in western boundary currents
Source: Sci Rep. 2017 Nov 6;7:14527. doi: 10.1038/s41598-017-14944-2 (PMC5674067; doi:10.1038/s41598-017-14944-2)

Half a century of temperature records reveal complex warming trends in coastal waters adjacent to western boundary currents

Nick T. Shears and Melissa M. Bowen

Supplementary material

Table S1. Cross correlation analysis between SST at coastal stations, and between SST and wind stress curl (WS), Southern Annual Mode (SAM), Southern Oscillation Index (SOI) and Pacific Decadal Oscillation Index (PDO) for each station. Based on detrended annual means. Bold values indicate significant correlations and red text indicate maximum significant correlations. Positive cross-correlations between annual SST and climate indices were not calculated as annual SST measurements at coastal stations do not influence future climate indices.

Lag (years)	SST			SST vs WS			SST vs SAM			SST vs SOI			SST vs PDO		
	Maria vs. Portobello	Maria vs. Leigh	Portobello vs. Leigh	Maria	Portobello	Leigh	Maria	Portobello	Leigh	Maria	Portobello	Leigh	Maria	Portobello	Leigh
-11	-0.118	-0.021	0.127	-0.062	0.214	0.141	0.14	0.021	-0.123	-0.061	0.194	0.201	-0.078	-0.023	-0.142
-10	-0.1	-0.156	0.178	-0.038	0.088	0.062	0.093	-0.034	0.023	-0.084	0.147	0.252	-0.15	0.014	-0.16
-9	-0.201	-0.125	-0.045	-0.052	-0.161	-0.22	0.018	-0.075	-0.15	-0.027	-0.099	-0.044	-0.054	-0.129	-0.216
-8	-0.034	-0.158	-0.07	-0.14	-0.138	-0.159	0.033	0.216	0.001	-0.136	-0.149	-0.104	-0.06	-0.09	-0.138
-7	-0.205	-0.158	-0.273	0.123	0.155	0.098	-0.091	0.006	-0.019	-0.076	0.039	-0.067	0.013	**-0.281**	-0.145
-6	-0.113	-0.066	-0.306	0.175	0.068	-0.005	-0.06	-0.074	0.029	0.085	0.089	-0.04	-0.027	-0.206	-0.071
-5	-0.055	-0.053	-0.165	**0.297**	-0.048	0.071	0.013	-0.204	0.043	0.114	0.013	0.033	-0.019	-0.135	-0.007
-4	0.215	**0.327**	0.044	0.131	0.015	0.142	-0.002	-0.112	-0.072	0.132	-0.124	-0.003	-0.076	0.06	-0.071
-3	**0.476**	**0.341**	0.105	**0.284**	-0.116	0.119	0.053	-0.12	0.023	**0.252**	-0.088	0.146	-0.133	0.185	0.026
-2	0.233	**0.332**	-0.007	0.143	0.035	0.065	-0.054	0.082	0.005	0.171	-0.078	-0.039	-0.085	0.133	0.05
-1	0.154	0.172	0.149	0.019	**0.279**	**0.358**	0.106	0.163	0.138	0.08	0.173	0.118	-0.232	-0.055	-0.114
0	0.237	**0.441**	**0.803**	**0.309**	**0.364**	**0.57**	0.103	0.134	0.25	**0.234**	**0.51**	**0.648**	-0.22	**-0.308**	**-0.376**
1	0.094	0.181	0.275	-	-	-	-	-	-	-	-	-	-	-	-
2	-0.152	-0.109	-0.169	-	-	-	-	-	-	-	-	-	-	-	-
3	-0.253	**-0.291**	-0.039	-	-	-	-	-	-	-	-	-	-	-	-
4	0.009	-0.17	-0.011	-	-	-	-	-	-	-	-	-	-	-	-
5	0.008	-0.113	-0.007	-	-	-	-	-	-	-	-	-	-	-	-
6	0.067	-0.133	-0.117	-	-	-	-	-	-	-	-	-	-	-	-
7	0.125	0.128	-0.042	-	-	-	-	-	-	-	-	-	-	-	-
8	0.259	0.219	0.09												
9	-0.014	0.028	-0.068	-	-	-	-	-	-	-	-	-	-	-	-
10	0.009	0.133	0.048	-	-	-	-	-	-	-	-	-	-	-	-
11	0.03	0.109	0.151	-	-	-	-	-	-	-	-	-	-	-	-

Table S2. Trend analysis of monthly climate variables over the long-term record and satellite era.

	Period	df	*T*	*p*
SOI	1946-2016	850	-0.72	0.474
	1982-2016	420	1.81	0.071
PDO	1946-2016	850	0.87	0.384
	1982-2016	420	-1.60	0.111
SAM	1957-2016	720	4.57	**<0.001**
	1982-2016	420	2.45	**0.015**
WS	1948-2016	828	6.85	**<0.001**
	1982-2016	420	3.67	**<0.001**

Figure S1. Seasonal cycle in sea surface temperature at Leigh, Portobello and Maria Island. Based on long-term monthly means. Dashed grey lines indicate minimum and maximum monthly means.

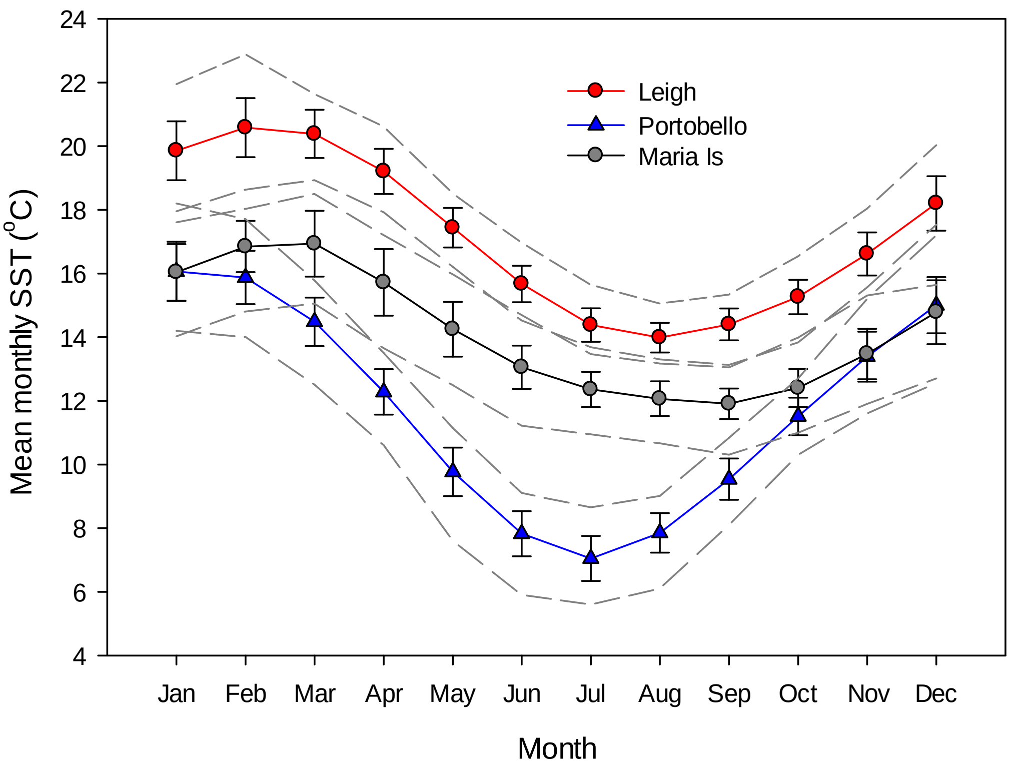

Figure S2. Transport stream function (in Sverdups, 1 Sv = 10^6 m^3/s) from the JRA-55 winds (1958-2016) show mean flow (upper panel) is counter-clockwise in the South Pacific along the contours, with amount of flow between contours equal to the difference in value between them. The trend in transport stream function at each location (lower panel) shows counter-clockwise flow increasing at southern latitudes. The white area shows the shallower submarine regions around New Zealand that extend to 33S and 55S that are treated as an island in the streamfunction integration. Map produced in Matlab R2017a, https://au.mathworks.com/products/matlab.html.

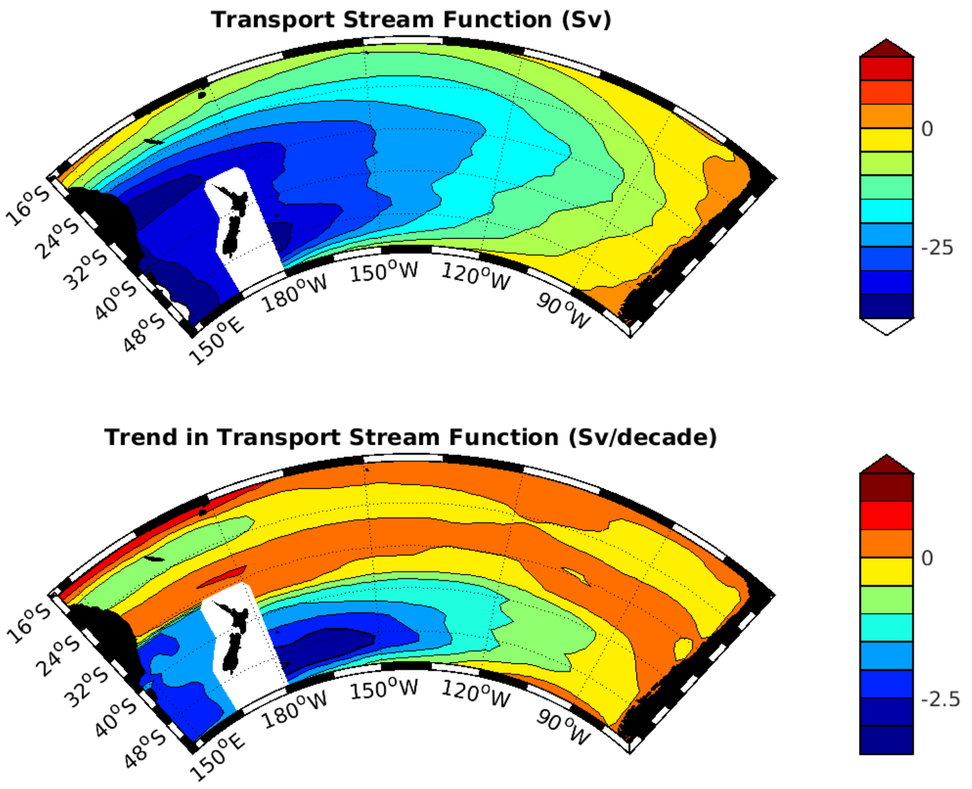

Figure S3. Long-term trends in climatic variables; wind stress curl (WS), Southern Annual Mode (SAM), Southern Oscillation Index (SOI) and Pacific Decadal Oscillation Index (PDO). Solid line shows 5 year moving average, black dashed line shows linear trend through complete time series and red dashed line show linear trend through the satellite era (1982-2016) (See Table S2 for trend analysis).

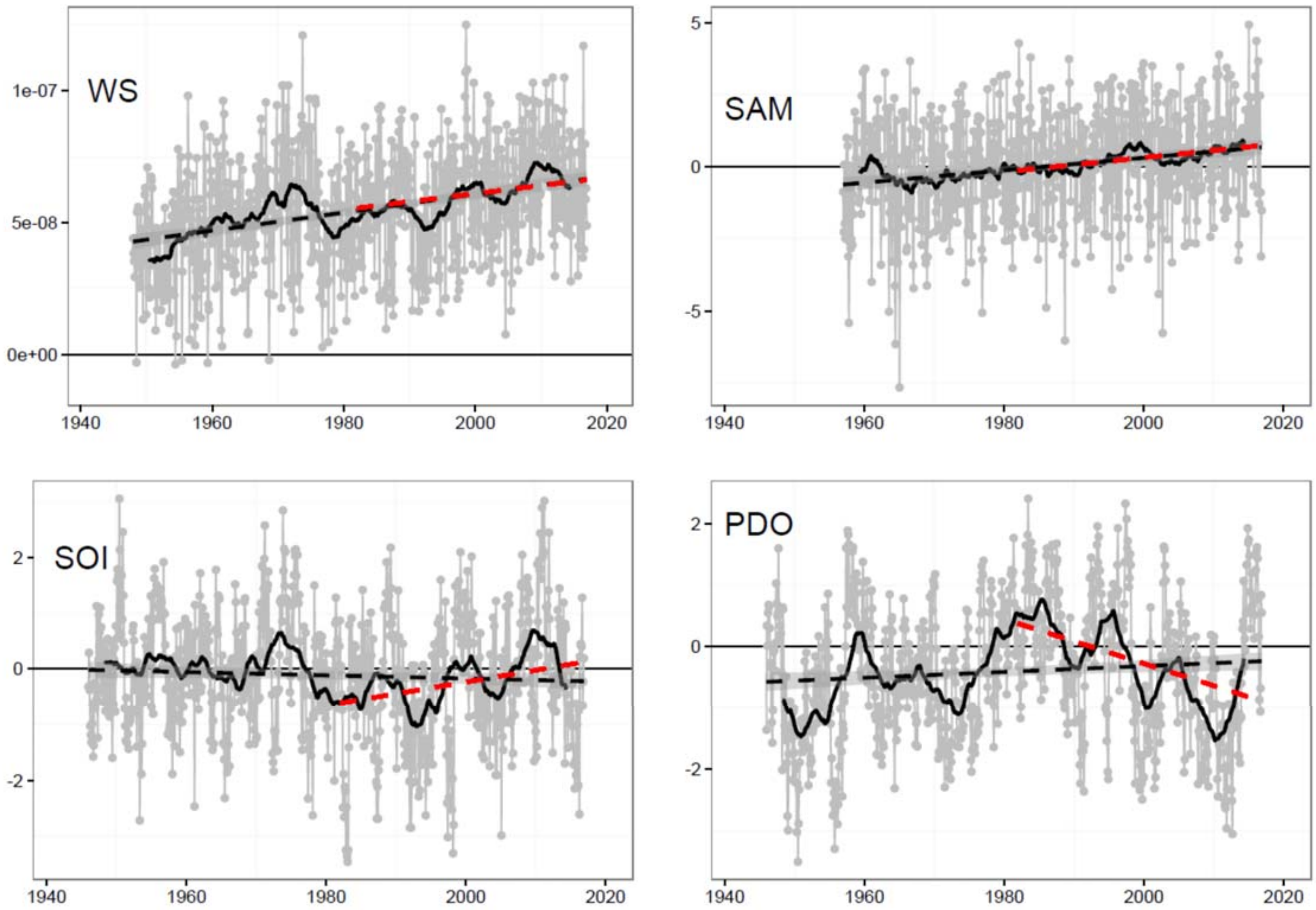

Figure S4. Seasonal variability in long-term change in sea surface temperature from 1967-2016 at each coastal station (confidence intervals that overlap with zero are not significant (α=0.05)). Slopes are based on linear regression of mean monthly SST anomalies analysed separately for each month over the last 50 years. Note that monthly measurements for each month are not available for every year at Maria Island (n = 33-39 years). The slope coefficient based on analysis of annual mean SST anomaly is also shown.

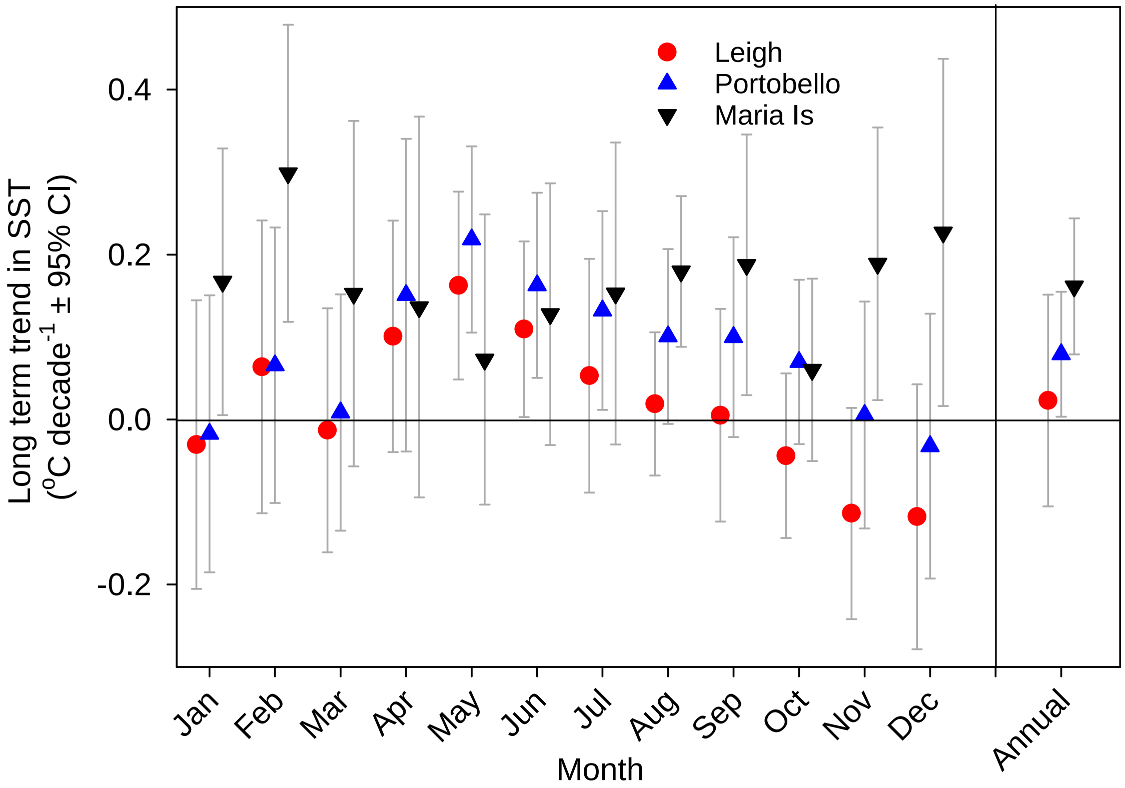

Figure S5. Trends in satellite derived SST [OISST] at offshore stations in each region (A) and in the three coastal SST records (B) from 1982-2016. Dashed line is linear trend (See Table S2 for trend analysis).

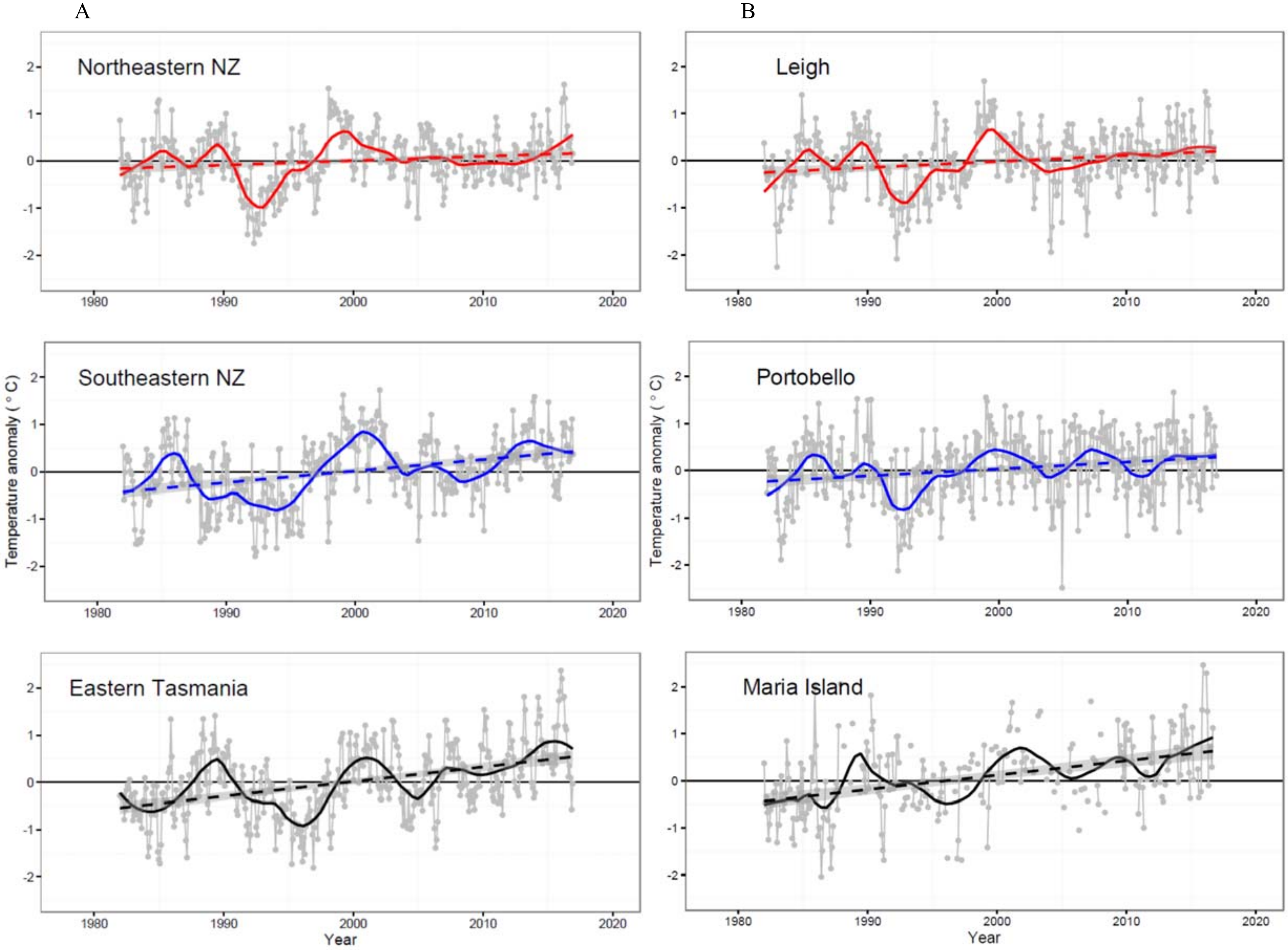

Figure S6. Seasonal variability in linear change in SST for offshore stations (OISST) in each region (A) and the coastal SST records (B) from 1982-2016. Slopes are based on linear regression of mean monthly SST anomalies. The slope coefficient based on analysis of annual mean SST anomaly is also shown.

A

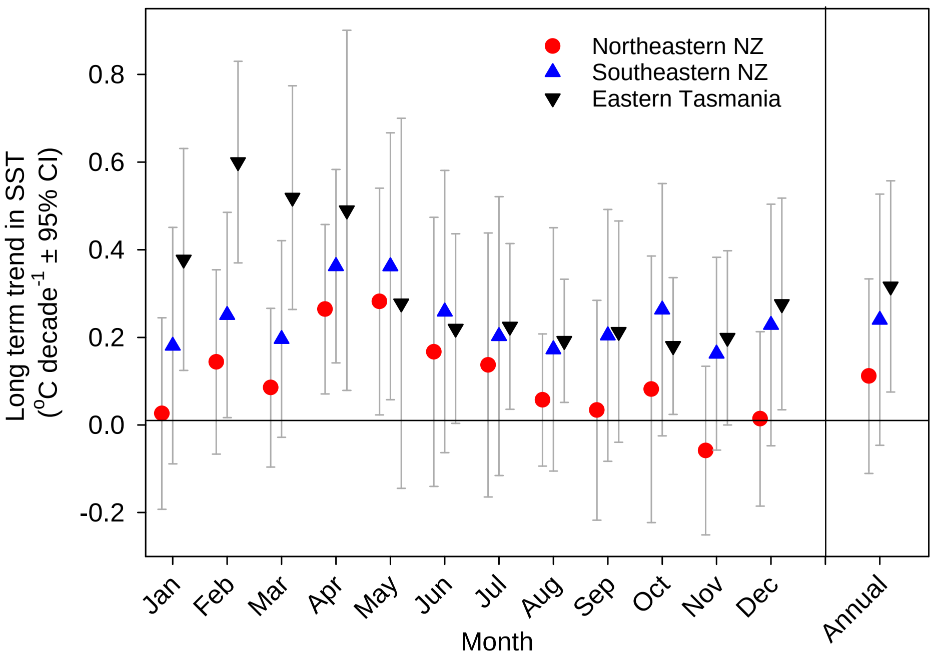

B

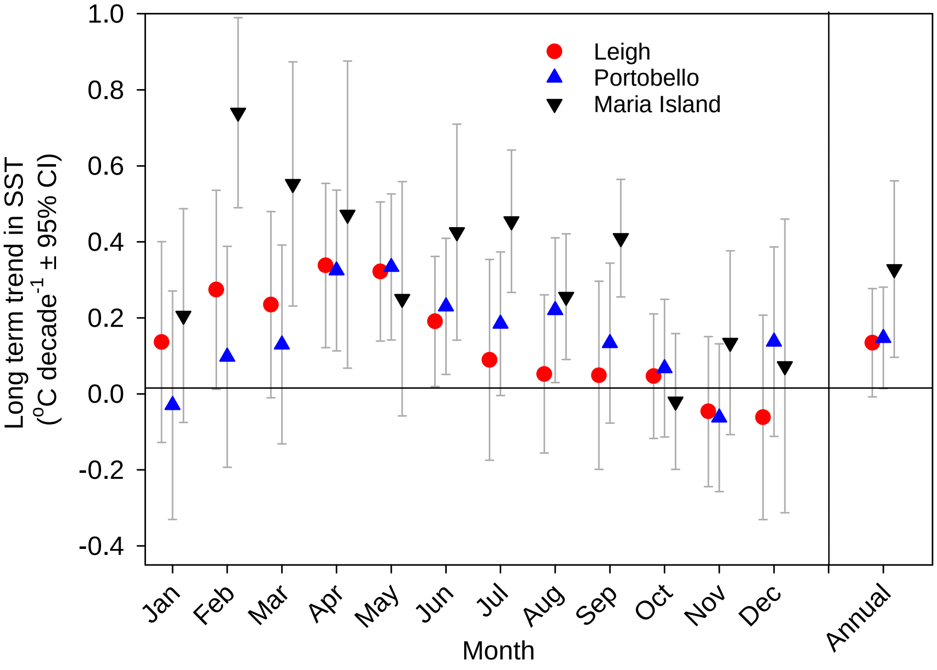

Figure S7. Trends in sea surface temperature in the southwest Pacific region from 1982-2016 based on globally reconstructed SST datasets: (A) ERSST v.4 and (B) HadISST. Grey dots denote locations with non-significant trends. Bathymetric contours are 500m and 1000m. Black dots indicate location of the three coastal SST stations. Maps produced in Matlab R2017a, https://au.mathworks.com/products/matlab.html.

A

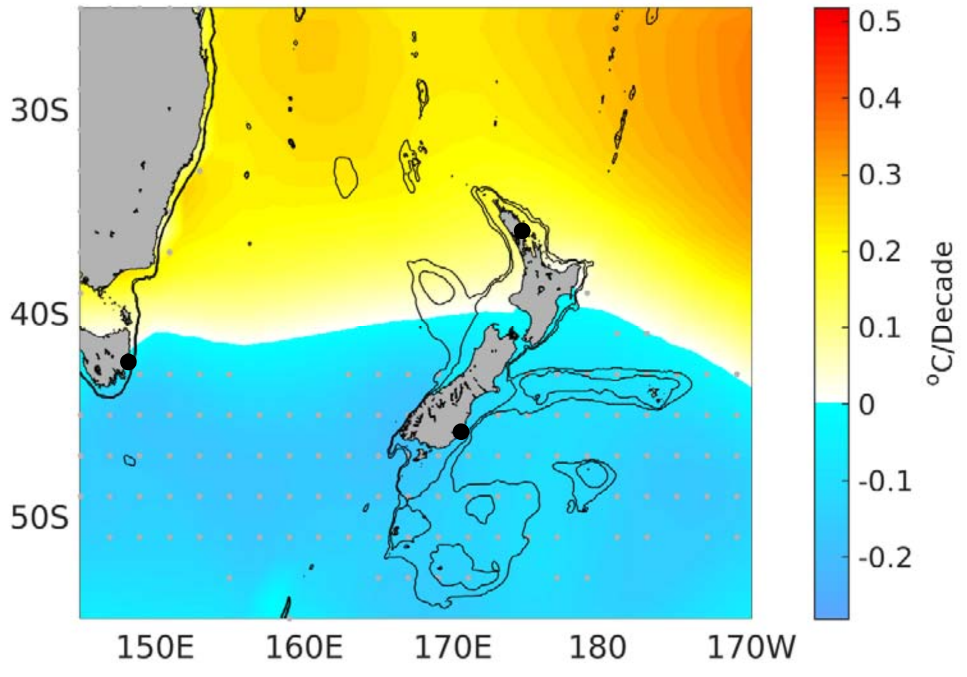

B

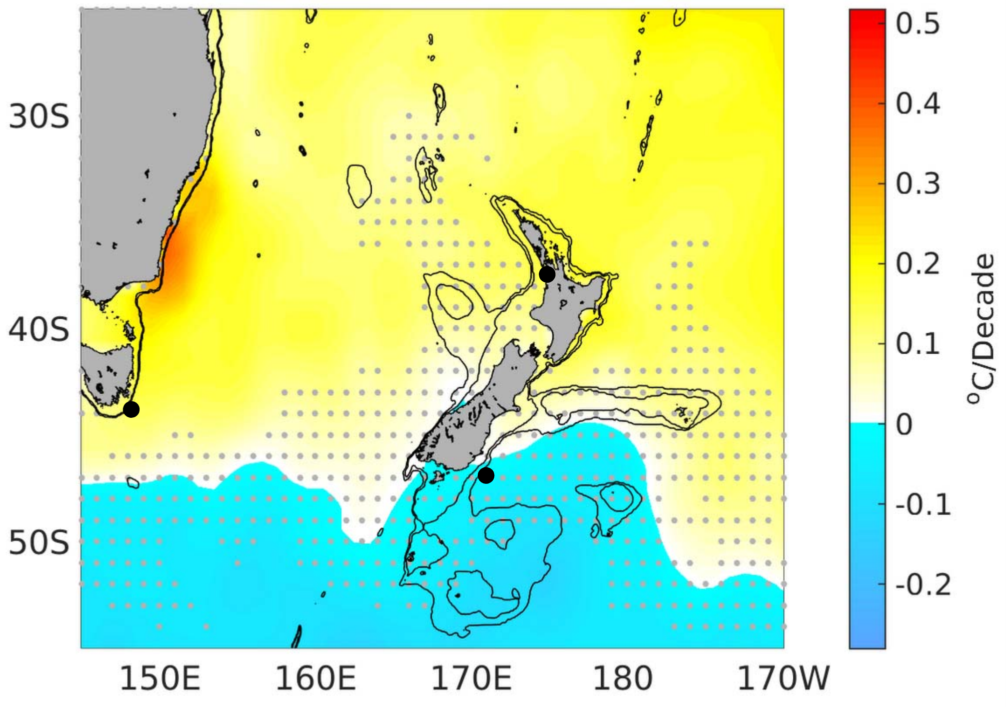

Supplement: Supplementary file 1 — Supplementary material [file 41598_2017_14944_MOESM1_ESM.pdf]
